# A geospatial dataset of urban infrastructure for emergency response in Portugal

**DOI:** 10.1016/j.dib.2023.109593

**Published:** 2023-09-18

**Authors:** João Paulo Just Peixoto, Daniel G. Costa, Paulo Portugal, Francisco Vasques

**Affiliations:** a IFBA, Federal Institute of Education, Science and Technology of Bahia, Valença, Brazil; b PPGM-UEFS, State University of Feira de Santana, Feira de Santana, Brazil; c INEGI, Faculty of Engineering, University of Porto, Porto, Portugal; d INESC-TEC, Faculty of Engineering, University of Porto, Porto, Portugal

**Keywords:** Urban emergencies, Urban planning, Hospital, Firefighters, Police, OpenStreetMap

## Abstract

Emergency response plays a critical role in mitigating the impact of disasters and ensuring public safety. Understanding a city's capability for emergency response is vital for effective disaster management and urban planning. This paper describes a comprehensive geospatial dataset that assesses the emergency response capability of cities in Portugal based on their urban infrastructure, accounting for the number of hospitals, police stations, fire department units, and metro/railway stations. These infrastructures are essential for attending to victims, mitigating emergency situations, and performing rescue operations. Besides that, the GeoJSON definitions of all Portuguese cities are also provided in the dataset, which were used to compute the number of the target facilities based on data from OpenStreetMap. The potential applications of this dataset are numerous, ranging from urban planning and resource allocation to disaster response strategy development. Moreover, it indicates where public investments are most required, especially when combined with others continuously updated public datasets with incidents in urban areas.

Specifications Table

**Table:** 

Subject	Earth and Planetary Sciences: Geographical Information System.
Specific subject area	This dataset contains information about the emergency response capability of cities across Portugal, based on existing urban infrastructure.
Data format	Raw, Analyzed, Filtered
Type of data	Table
Data collection	Data was obtained as shapefiles from DGT, an official institution from Portugal for cartographic data. The shapefiles were mixed in the QGIS tool and then exported as a single GeoJSON file. We then wrote two Python scripts to split this GeoJSON file into several GeoJSON files (one for each city). In the sequence, OpenStreetMap data was gathered for each city, using the Overpass API. Finally, the second script computed the number of Points of Interest in each city from the OpenStreetMap data obtained from the Overpass API.
Data source location	Shapefile data of Portuguese cities comes from an official agency (https://www.dgterritorio.gov.pt/cartografia/cartografia-tematica/). Metadata of urban infrastructure comes from OpenStreetMap (https://www.openstreetmap.org/).
Data accessibility	Repository name: Mendeley Data Data identification number: DOI: 10.17632/jg7xxd524s.4 Direct URL to data: Just Peixoto, João Paulo; Costa, Daniel G.; Portugal, Paulo; Vasques, Francisco (2023), “A Geospatial Dataset of Urban Infrastructure for Emergency Response in Portugal”, Mendeley Data, V4, doi: 10.17632/jg7xxd524s.4[1]

## 1 Value of the Data

• The usefulness of the presented dataset lies in the numerical assessment of critical urban infrastructure that is not typically associated, but that impact the effective perception of resilience and public safety in urban areas. As an important member of the European Union that is committed with urban development programs and EU Horizon initiatives, Portugal has important urban development challenges that can be supported by public databases as this one.
• Understanding a city's emergency response capability is crucial for effective disaster management. This dataset offers valuable insights into the dispersion and density of essential infrastructure namely hospitals, police stations, fire department units, and metro/railway stations in Portugal. This provided numerical perception, even though not directly presenting distribution fairness and spatial coverage details, is valuable when giving a broader perspective of smaller and larger urban areas.
• By identifying gaps or areas of strength, this dataset assists in the development and refinement of disaster management strategies for Portuguese cities. For the smart cities revolution as promoted by the UN Agenda 2030 and also by the European Commission, knowing the current emergency-related urban infrastructure is a very relevant asset.
• This presented dataset can be easily integrated with other public datasets as the ones provided by the Eurostat statistical authority.

## 2 Objective

Emergencies in cities pose significant challenges, with their negative impacts reverberating throughout society when critical situations are not properly handled. Typically, a deeper understanding of urban infrastructure can play a crucial role in mitigating these negative effects [2,3], with geo-referenced systems emerging to provide more comprehensive knowledge about the city's resilience and safety [4]. Moreover, a better understanding of urban infrastructure allows for the implementation of proactive measures, such as strengthening critical structures and establishing robust communication networks, usually performed by well-informed public agents.

By harnessing the power of urban infrastructure knowledge, cities can enhance their preparedness and response capability, ultimately safeguarding their residents and fostering long-term resilience. However, the challenge has been to provide reliable public datasets that can be exploited by different approaches, from traditional urban planning [5] to smart city initiatives [6].

When properly structured, urban areas can enable faster and more efficient mitigation and rescue operations, minimising casualties and saving lives.

## 3 Data Description

The dataset described in this paper is composed of four different groups of data, described as follows:

• 308 records of metadata about the urban resilience of Portuguese cities when concerning their capability to respond to critical situations, all in the GeoJSON format. These records comprise both continental Portugal and its archipelagos (Azores and Madeira).
• 308 records of the same cities but now in the OpenStreetMap XML file format (OSM), supporting different types of data visualisation and processing.
• 1 full GeoJSON file comprising the whole Portugal (continental and archipelagos).
• 1 CSV file that summarises the number of each type of Point of Interest (PoI) in each city.

The entire dataset is composed of 618 files organised in different directories according to their types. They provide some type of information of Portugal (territorial borders) and of all its 308 cities. Data of the cities were collected from the Portuguese government [7], being originally represented in the form of a shapefile containing the polygons that comprise the entire area of every city. The shapefile was converted to its GeoJSON correspondence, split in several files (one for each city), and then used by a Python script to gather OpenStreetMap data from the Overpass API [8]. The OpenStreetMap data for each city contains the GPS coordinates of every Point of Interest considered in this dataset: hospitals, fire stations, police stations, and metro/railway stations. In this context, a Point of Interest is defined as any facility that may have some role in an emergency mitigation procedure [9]. Actually, it is considered herein the official municipal administrative division in Portugal, resulting in 308 municipalities (“concelhos”), which may contain both urban and rural areas. For the sake of homogeneity, and also considering that rural emergencies will be typically supported by nearby urban infrastructure, all 308 municipalities will be referred to in this dataset as a city.

Table 1 presents the features contained in the GeoJSON files of the cities. The OSM files and the GeoJSON of entire Portugal follow standard configurations and thus they will be not described here.

**Table 1:** The GeoJSON metadata for each city.

Feature	Description
NAME_1	The name of the city
Hospitals	The number of hospitals
fire_stations	The number of fire stations
police_stations	The number of police stations
railway_stations	The number of railway stations, including metro and urban trains
total_pois	The sum of all PoIs
types_of_pois	The number of different types of PoIs in the city (from 0 to 4)
Geometry	The geometry of the city, which is defined by the polygon that forms its perimeter (list of the GPS coordinates of all vertices)

The features of the CSV file are presented in Table 2 with each feature as a column in the defined table.

**Table 2:** The CSV file with summarised information of the cities.

Feature	Description
City	The name of the city
Hospitals	The number of hospitals
Fire stations	The number of fire stations
Police stations	The number of police stations
Railway stations	The number of railway stations, including subways and urban trains
Total PoIs	The sum of all Points of Interest
Types of PoIs	The number of different types of PoIs in the city (from 0 to 4)

## 4 Experimental Design, Materials and Methods

**Fig. 1:**
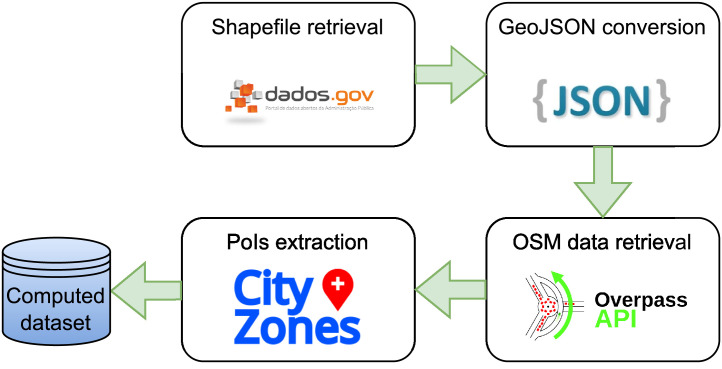
Dataset creation methodology.

The process of data collection and processing is depicted in Fig. 1. This overall workflow can be replicated for other cities, taking advantage of the described pre-processing steps and some functionalities provided by our CityZones tool described in [9].

The creation of the dataset involved the use of some software tools for data conversion and extraction. For shapefile conversion into the GeoJSON format, QGIS was used [10]. QGIS is an open-source Geographic Information System, widely used for geographic data creation and analysis. This tool allowed an easy conversion of the data into a more suitable format for further processing. After having a GeoJSON file for the entire Portugal, a Python 3 script was written to split the full GeoJSON file into several files, one for each city. This approach was important so the following steps could be performed and verified without the need to wait for the processing of the entire country data. Also, by splitting into several files, it might allow optimised analysis on specific cities in future works.

Having separated files for each city, another Python script was used to retrieve data (in the OSM format) from the Overpass API. Overpass API is an open web-based API that allows OpenStreetMap data retrieval using its own query language. By using the OSMPoIs Python library defined in [11], which connects to the Overpass API, it is possible to request OSM data for a specific perimeter just passing the polygon that defines the city's perimeter as a parameter. For our dataset creation, the API returns all the PoIs within the boundaries of the polygon provided. Finally, with an OSM file for each city, the OSMPoIs library was used in the same Python script to extract the Points of Interest of every city, then writing the number of each type of PoI into the corresponding GeoJSON file.

Overall, this dataset makes it possible to perform different types of analysis regarding the geographic data of the cities of Portugal and their emergency response centres. The existence of single files for each city is appropriate when working with only one or some of them, while the full GeoJSON file provides a complete aggregation that allows major perspectives of the country. In this sense, we envision a great number of practical uses of this dataset, benefiting areas in the scopes of urban planning, public policies making, and smart cities designing.

**Fig. 2:**
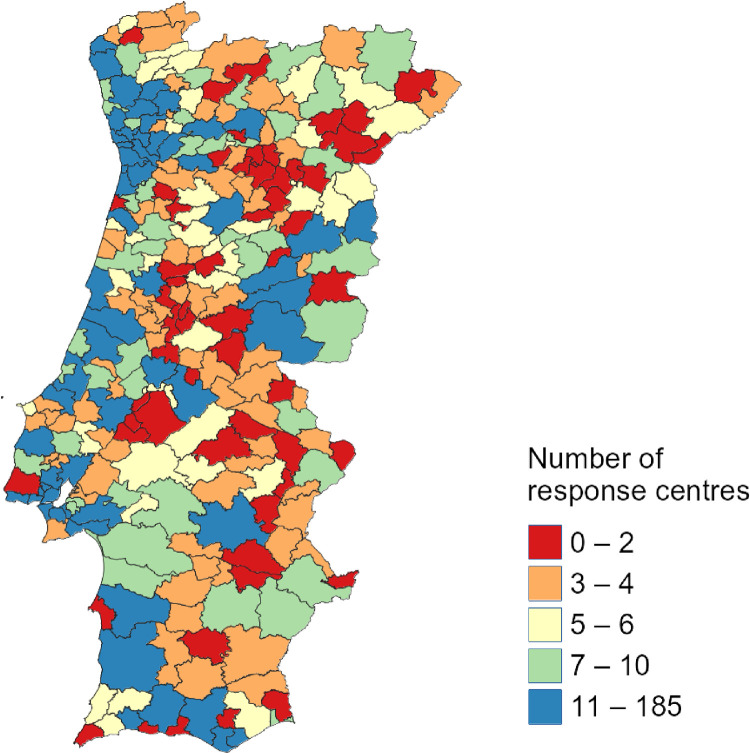
The total number of response centres in each city in mainland Portugal.

Let's consider a practical example to illustrate an expected usage. We can examine the distribution of response centres across continental Portugal by leveraging the QGIS tool and this dataset, generating informative maps. Fig. 2 classifies every city in continental Portugal regarding the number of response centres.

A city painted in red means it has at most two response centres at total, while blue cities are the ones with most response centres (minimum of 11). The values were distributed equally in each class, with the number of cities on each colour group being roughly the same.

**Fig. 3:**
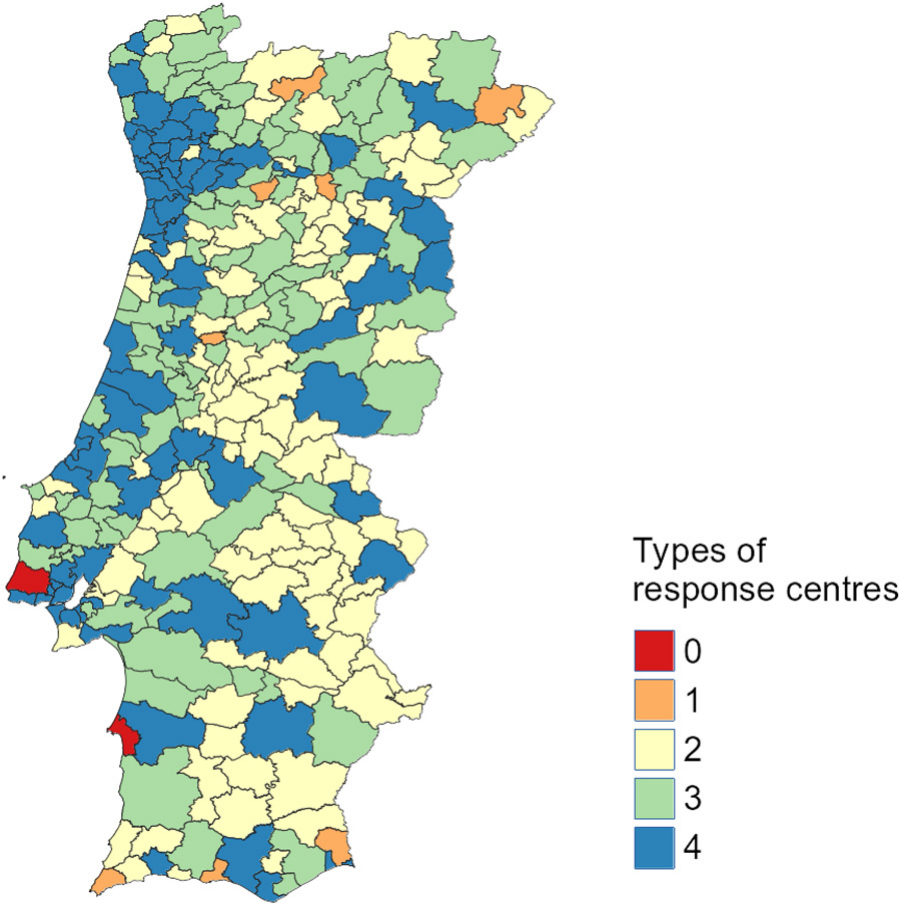
Number of types of response centres in each city of mainland Portugal.

In a second analysis, Fig. 3 classifies the cities regarding the number of different types of response centres within their delimited areas. In this case, what matters is how many different types of PoIs each city has, varying from 0 to 4.

While this is just a usage example of the dataset, these two maps can give a broader picture of the distribution of response centres in mainland Portugal, demonstrating the concentration of PoIs in higher urbanised and richer areas. Nevertheless, many other types of analyses are possible, such as fairness in PoI distribution (how balanced is the PoI distribution within each city), relation of wildfires with the presence of fire departments, number of disasters and incidents and their relationship with emergency-related infrastructure, among others. In fact, the metadata present in the GeoJSON files will allow any researcher to make different classifications regarding the number of PoIs by using any GIS software, bringing valuable results.

## Limitations

Not applicable.

## Ethics Statement

This work does not involve human subjects, animal experiments, or any data collected from social media platforms.

## CRediT authorship contribution statement

**João Paulo Just Peixoto:** Conceptualization, Methodology, Software, Validation. **Daniel G. Costa:** Conceptualization, Methodology, Writing – review & editing, Supervision. **Paulo Portugal:** Validation, Writing – review & editing. **Francisco Vasques:** Validation, Writing – review & editing.

## Data Availability

emergency_portugal (Original data) (Mendeley Data)
